# Identification of Novel Surface-Exposed Proteins of *Rickettsia rickettsii* by Affinity Purification and Proteomics

**DOI:** 10.1371/journal.pone.0100253

**Published:** 2014-06-20

**Authors:** Wenping Gong, Xiaolu Xiong, Yong Qi, Jun Jiao, Changsong Duan, Bohai Wen

**Affiliations:** State Key Laboratory of Pathogen and Biosecurity, Beijing Institute of Microbiology and Epidemiology, Beijing, China; University of Texas Medical Branch, United States of America

## Abstract

*Rickettsia rickettsii*, the causative agent of Rocky Mountain spotted fever, is the most pathogenic member among *Rickettsia* spp. Surface-exposed proteins (SEPs) of *R. rickettsii* may play important roles in its pathogenesis or immunity. In this study, *R. rickettsii* organisms were surface-labeled with sulfo-NHS-SS-biotin and the labeled proteins were affinity-purified with streptavidin. The isolated proteins were separated by two-dimensional electrophoresis, and 10 proteins were identified among 23 protein spots by electrospray ionization tandem mass spectrometry. Five (OmpA, OmpB, GroEL, GroES, and a DNA-binding protein) of the 10 proteins were previously characterized as surface proteins of *R. rickettsii*. Another 5 proteins (Adr1, Adr2, OmpW, Porin_4, and TolC) were first recognized as SEPs of *R. rickettsii* herein. The genes encoding the 5 novel SEPs were expressed in *Escherichia coli* cells, resulting in 5 recombinant SEPs (rSEPs), which were used to immunize mice. After challenge with viable *R. rickettsii* cells, the rickettsial load in the spleen, liver, or lung of mice immunized with rAdr2 and in the lungs of mice immunized with other rSEPs excluding rTolC was significantly lower than in mice that were mock-immunized with PBS. The *in vitro* neutralization test revealed that sera from mice immunized with rAdr1, rAdr2, or rOmpW reduced *R. rickettsii* adherence to and invasion of vascular endothelial cells. The immuno-electron microscopic assay clearly showed that the novel SEPs were located in the outer and/or inner membrane of *R. rickettsii*. Altogether, the 5 novel SEPs identified herein might be involved in the interaction of *R. rickettsii* with vascular endothelial cells, and all of them except TolC were protective antigens.

## Introduction

*Rickettsia rickettsii* is an obligate intracellular Gram-negative bacterium that causes Rocky Mountain spotted fever (RMSF), a serious life-threatening disease. RMSF was first found in the Snake River Valley of Idaho in 1896 and described by Edward E Maxey [1]. Patients suffering from RMSF usually present fever, headache, myalgias, and rash, as well as a history of tick bite or contact. For serious *R. rickettsii* infection, patients will develop symptoms of acute lung edema, renal failure, and/or encephalitis [2], [3] due to wide spread vasculitis caused by rickettsial infection of endothelial cells lining the small blood vessels in these vital organs [4], [5].

A “zipper-like” invasion strategy has been suggested for rickettsial invasion of non-phagocytic host cells [6], [7], whereby a receptor-mediated mechanism is initiated when a rickettsial protein induces host intracellular signaling through extracellular stimulation of a receptor on the surface of host cells [8]. This mechanism suggests that surface-exposed proteins (SEPs) of both rickettsiae and host cells play fundamental roles in the interactions between rickettsia and the host cell.

Surface cell antigen (Sca) family proteins are recognized as the major SEPs of rickettsiae [9], playing important roles in rickettsial pathogenesis. Both Sca0 (outer membrane protein A, OmpA) and Sca1 are involved in rickettsial attachment to host cells [10], [11]. Sca5 (OmpB) is associated with rickettsial adherence to and invasion of host cells [12]. Sca2 functions as a formin mimic that causes actin-based motility of rickettsiae in host cells [13], and Sca4 activates vinculin and interacts with the actin cytoskeleton of host cells [14].

In addition to their functions in pathogenesis, SEPs are likely to be important in activating immune cells to elicit protective responses against rickettsial infection. OmpA and OmpB are well known protective antigens of spotted fever group (SFG) rickettsiae [15]. Recently, a surface protein (YbgF) of *R. heilongjiangensis* has been recognized as a protective antigen [16].

Proteomics analysis of rickettsial surface proteins has the potential to scientifically identify surface proteins, including those required for the interaction of rickettsiae with host cells to cause infection and those required for induction of protective immune responses against the infection. In the present study, proteomic analysis of surface cell proteins was used to identified 10 major SEPs of *R. rickettsii*, 5 of which were first recognized as the surface proteins of *R. rickettsii* and found to be associated with its outer and/or inner membrane by immuno-electron microscopic assay. The genes encoding the novel SEPs of *R. rickettsii* were expressed in prokaryotic cells, and the resultant recombinant SEPs (rSEPs) were used to immunize mice to evaluate their immunoprotective efficacies. The sera from mice immunized with each of the 5 rSEPs were used in an *in vitro* serum neutralization test to assay the interaction of the surface molecules with host cells.

## Materials and Methods

### Ethics Statement

Female C3H/HeN mice at 6 weeks of age were obtained from Vital River Laboratories (Beijing, China). The use of animals in the present study was approved by the Institute of Animal Care and Use Committee (IACUC No: AMMS-2013-009) at Academy of Military Medical Science (AMMS). All of the facilities were accredited by the AMMS Animal Care and Ethics Committee, and the animal care met the standard of the committee. All efforts were made to minimize suffering of the animals.

### Strains and plasmids

*Rickettsia rickettsii* (Sheila Smith strain) were grown in Vero cells cultured in Dulbecco's modification of Eagle medium (DMEM) (Hyclone, Beijing, China) supplemented with 5% fetal bovine serum (FBS) (Hyclone, San Jose, CA). The number of *R. rickettsii* or viable rickettsial organisms in suspension was detected by qPCR specific for *R. rickettsii* [17] and plaque assay [18], respectively. *Escherichia coli* (*E. coli*) BL21 (Novagen, Madison, WI) was cultured in Luria-Bertani (LB) medium for expression of recombinant proteins. The prokaryotic expression plasmid pET32a (+) (Novagen, Madison, WI) was used as a vector for expression of target genes.

### Biotinylated surface-exposed proteins

*Rickettsia rickettsii* was cultured in Vero cells and then the propagated organisms were purified from the host cells by isopycnic density gradient centrifugation according to a previous method [19]. The SEPs of the purified rickettsiae were biotinylated with sulfo-NHS-SS-biotin (Thermo Science, Rockford, IL) and affinity-purified with streptavidin-agarose as described previously [16]. The protein concentration in the rickettsial lysate suspension was determined using Bradford Protein Assay Kit (Real Times, Beijing, China). Eluted biotinylated proteins (300 µg) were treated with 2-D Clean Up Kit (GE healthcare, Waukesha, WI) and then separated by isoelectric focusing electrophoresis with a pH 3-10 NL IPG strip (Bio-Rad, Richmond, CA) followed by 12% SDS-PAGE as described previously [20]. The protein spots observed following 2D-PAGE were stained with Silver Strain Plus Kit (Bio-Rad, Richmond, CA).

### Mass spectrometry and bioinformatics analysis

Following 2D-PAGE, isolated protein spots were digested with trypsin and identified by electrospray ionization tandem mass spectrometry (ESI-MS/MS), which was performed by the National Center of Biomedical Analysis (Beijing, China). Mass fingerprints of the peptides detected by ESI-MS/MS were compared against the National Center for Biotechnology Information (NCBI) non-redundant databases using the Mascot search engine (http://www.matrixscience.co.uk) [20]. The signal peptides of the identified SEPs were analyzed using SignalP4.1 web server (http://www.cbs.dtu.dk/services/SignalP/), and transmembrane beta-strands of Gram-negative bacteria outer membrane proteins were predicted using PRED-TMBB web server (http://bioinformatics.biol.uoa.gr/PRED-TMBB) [21], [22]. The SignalP 4.1 server predicts the presence and location of signal peptide cleavage sites in amino acid sequences from different organisms. The method incorporates a prediction of cleavage sites and a signal peptide/non-signal peptide prediction based on a combination of several artificial neural networks. The prediction of the transmembrane strands and the topology of beta-barrel outer membrane proteins were performed with the PRED-TMBB web server based on a Hidden Markov Model (HMMs) and according to the conditional maximum likelihood criterion. The SEPs families were classified with Pfam-A using the Pfam database (http://pfam.janelia.org/).

### Preparation of recombinant proteins

The target gene sequences were amplified according to the genomic sequence of *R. rickettsii* (GenBank accession number: CP000848) using polymerase chain reaction (PCR) with cognate primer pairs (Table S1). Each PCR-amplified gene fragments was inserted into the pET32a (+) plasmid, and *E. coli* cells were transformed with each recombinant plasmid. Recombinant proteins were purified from the *E. coli* lysate using Ni-NTA affinity resin (Qiagen GmbH, Hilden, Germany) according to the manufacture's instruction.

Fifteen micrograms of each purified recombinant protein was subjected to SDS-PAGE for electrophoresis and then transferred to polyvinylidene fluoride (PVDF) membranes (Millipore, Billerica, MA) for immunoblotting. The membranes were immunoblotted with sera collected from mice at 28 days after infection with *R. rickettsii* [23]. Finally, the protein bands in the PVDF membrane were developed with DAB Developing Kit (Boster, Wuhan, China) according to the manufacture's instruction.

### Immunization of mice

Seven groups of mice (5 mice per group) were immunized with each of 5 recombinant proteins, whole cell antigens (WCA, inactivated at 90°C for 20 min [19]) of *R. rickettsii* (positive control), or PBS (negative control). Briefly, each mouse was injected subcutaneously with 30 µg of antigen in 200 µl PBS or PBS alone mixed with complete Freund's adjuvant (CFA, Sigma-Aldrich, MO). On days 28 and 42 after primary immunization, each mouse was injected intraperitoneally with 20 µg of cognate antigen in 200 µl PBS or PBS alone mixed with incomplete Freund's adjuvant (IFA, Sigma-Aldrich, MO). Blood samples were collected at day 56 after first immunization of mice to prepare immune sera.

### Serum neutralization test

To host *R. rickettsii*, we used cells from a human endothelial hybrid cell line (EA.hy 926, ATCC) cultured in DMEM containing 10% heat-inactivated FBS. Each type of immune serum was heated (56°C for 30 min) and filter sterilized [24]. Then, 150 µl of each serum was mixed with *R. rickettsii* cells in 150 µl of DMEM (1.0 ×10^6^ PFU/ml) at room temperature for 60 min. Subsequently, the serum-rickettsial mixture was added to 9.7×10^5^ host cells in 2.7 ml of DMEM containing 1% heat-inactivated FBS. This mixture was divided into 3 replicate wells in a 24-well plate (Corning, Corning, NY) and cultured at 33°C for 6 h [24]. After removing the supernatant, the remaining cells in each well were collected for DNA extraction with DNeasy Blood & Tissue Kit (Qiagen GmbH, Hilden, Germany). The DNA samples were evaluated by quantitative polymerase chain reaction (qPCR) with primers specific for *R. rickettsii* [25].

### Mice infection

Fifteen days after the last immunization, each of the vaccinated mice was challenged i.p. with a sublethal dose of *R. rickettsii* (6×10^6^ PFU). Five days later, the infected mice were sacrificed and their spleens, livers, and lungs were collected. Next, DNA was extracted from 20 mg of tissue of each organ for *R. rickettsii*-specific qPCR [25].

### Detection of surface-exposed proteins by immuno-electron microscopic assay

For immuno-electron microscopic analysis [26], *R. rickettsia*-infected Vero cells were treated with fixing agent (4% paraformaldehyde, 0.5% glutaraldehyde, 0.3% picric acid, 0.1M sodium cacodylate, pH 7.4) for 4 h on ice. Then, the cells were sequentially dehydrated with 50%, 70%, 85%, and 95% alcohol and successively permeated in a mixture of LR White (Spi Supplies, West Chester, PA) and alcohol or LR White alone according to the standard method. The samples were embedded in Spi-Pon 812 resin (Spi Supplies, West Chester, PA) and transferred to 200 mesh nickel gird (BeiJingZhongXingBaiRui Technology Co., ltd, Beijing, China). The grids were then incubated with each immune serum (1∶10 dilution) for 2 h. After washing with blocking buffer, the girds were incubated with a goat anti-mouse IgG labeled with 10 nm colloidal gold particles (Aurion, EMS) (1∶20 dilution) for 2 h at room temperature. Following washing, the grids were fixed in 1% glutaraldehyde for 10 min, washed, and stained with uranyl acetate (Spi Supplies, West Chester, PA) and lead citrate (Spi Supplies, West Chester, PA). Finally, the girds were examined using a transmission electron microscopy (TEM) at 80 kV (H-7650, Hitachi Chemical co., Ltd, Japan).

### Statistical analysis

All statistics were computed using SAS statistical software (version 9.1, SAS Institute Inc., Cary, NC). The statistical significances of the differences in rickettsial numbers produced by qPCR were assayed using the *T* test or Wilcoxon two-sample test according to their normality and homogeneity of variance. *P*<0.05 was considered significantly different.

## Results

### Identification of surface-exposed proteins of *R. rickettsii*

**Figure 1:**
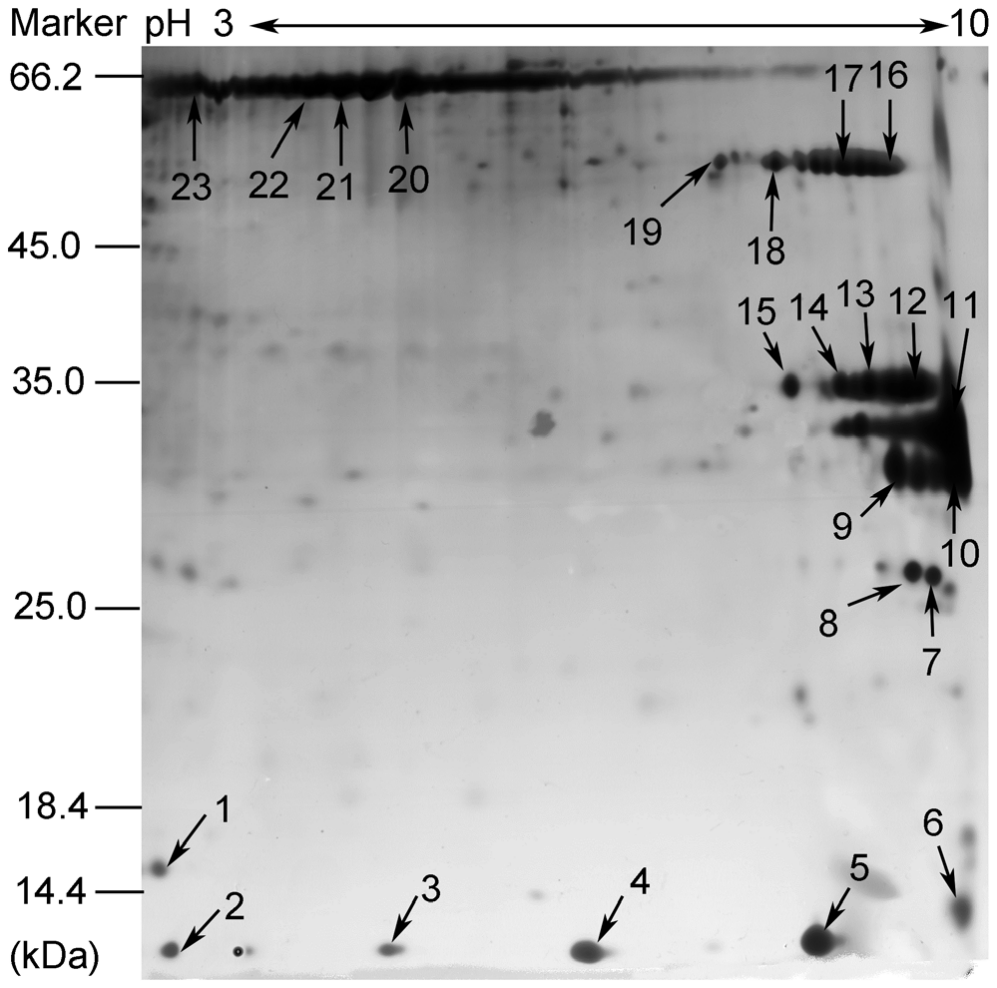
Profile of surface-exposed proteins in *R. rickettsii*. The isolated biotinylated proteins (300 µg) of *R. rickettsii* were separated by isoelectric focusing electrophoresis with a pH 3–10 strip followed by 12% SDS-PAGE. The protein spots in the gel were numbered from 1 to 23 and subjected to ESI-MS/MS analysis. The relative molecular masses of the marker proteins are indicated in kDa on the left side of the figure and the proteins identified by ESI-MS/MS are listed in Table 1.

After 2D-electrophoresis of the biotinylated proteins of *R. rickettsii*, 23 protein spots (indicated by numbers 1 to 23) were deeply colored on the gel after staining (Figure 1). Ten proteins were identified from the 23 protein spots by mass spectrometry and bioinformatics analysis. Five proteins (OmpA, OmpB, GroEL, GroES, and a DNA-binding protein) had been previously described as membrane-associated surface proteins of *R. rickettsii*. The other 5 proteins (Adr1, Adr2, OmpW, Porin_4, and TolC) were identified as surface proteins of this pathogen.

After submission of the protein sequences to the SignalP 4.1 web server, 6 proteins (Adr1, Adr2, OmpB, OmpW, Porin_4, and TolC) were predicted to carry a N-terminal signal peptide. The remaining proteins (OmpA, GroEL, GroES, and DNA-binding protein) did not contain a signal peptide (Table 1).

**Table 1:** Biotinylated surface-exposed proteins of *R. rickettsii* analyzed by ESI-MS/MS.

Spot NO	Annotation	Accession numbers^a^	Signal peptide^b^ (position)	Scores for β-barrel^c^	MW(Da)	Score^d^	Predicted function	Pfam family^e^
10,11	Adr1	A1G_07045	YES (22,23)	2.818	26486	369	Cell envelope biogenesis, outer membrane	PF12393
7,8	Adr2	A1G_07050	YES (22,23)	2.836	24083	355	Cell envelope biogenesis, outer membrane	PF12393
1	DNA-binding protein	A1G_06785	NO	2.979	16102	115	Adsorption capacity and tendency to small nucleotide fragments	PF00210
20,21,22,23	GroEL	A1G_05320	NO	2.886	58590	1133	60 kDa heat shock protein (Hsp60)	PF00118
2,4,5,6	GroES	A1G_05325	NO	3.049	10515	495	10 kDa heat shock protein (Hsp10)	PF00166
17,18,19	Porin_4	A1G_00605	YES (19,20)	2.882	48023	1084	Molecular sieve	PF13609
12	OmpA	A1G_06990	NO	2.873	224236	211	Invasion and adhesion	PF03797
13,14,15	Omp B	A1G_06030	YES (29,30)	2.868	168082	387	Invasion and adhesion	PF03797
9	OmpW	A1G_00640	YES (23,24)	2.911	26509	140	Transportation of hydrophobic material across cell membrane	PF03922
16	TolC	A1G_01745	YES (19,20)	2.916	49691	90	Outer membrane efflux proteins	PF02321

a The National Center for Biotechnology Information (NCBI, http://www.ncbi.nlm.nih.gov/). Data were retrieved on March 4, 2013.

b The signal peptide was predicted using SignalP 4.1 web server (http://www.cbs.dtu.dk/services/SignalP/). Websites were accessed on March 4, 2013.

c The transmembrane strands and the topology of beta-barrel outer membrane proteins of *R. rickettsii* were predicted using the PRED-TMBB web server (http://bioinformatics.biol.uoa.gr/PRED-TMBB), which is based on a Hidden Markov Model, trained according to the Conditional Maximum Likelihood criterion. A score less than 2.965 indicated that the protein may be a membrane protein. Data were retrieved on March 7, 2013.

d The ions score is presented as -10*Log (P), where P is the probability that the observed match is a random event. Individual ions scores >54 indicate identity or extensive homology (*P*<0.05). Protein scores are derived from ions scores on a non-probabilistic basis for ranking protein hits.

e The Pfam-A was used to predict the family of the protein, Pfam does not allow any amino-acid to match more than one Pfam-A family(http://pfam.sanger.ac.uk/), unless the overlapping families are part of the same clan. In cases where two members of the same clan match the same region of a sequence, only one match is show, that with the lowest E-value. The E-value cut-off in Pfam-A search was set to 1.0. Data were retrieved on July 19, 2013.

**Figure 2:**
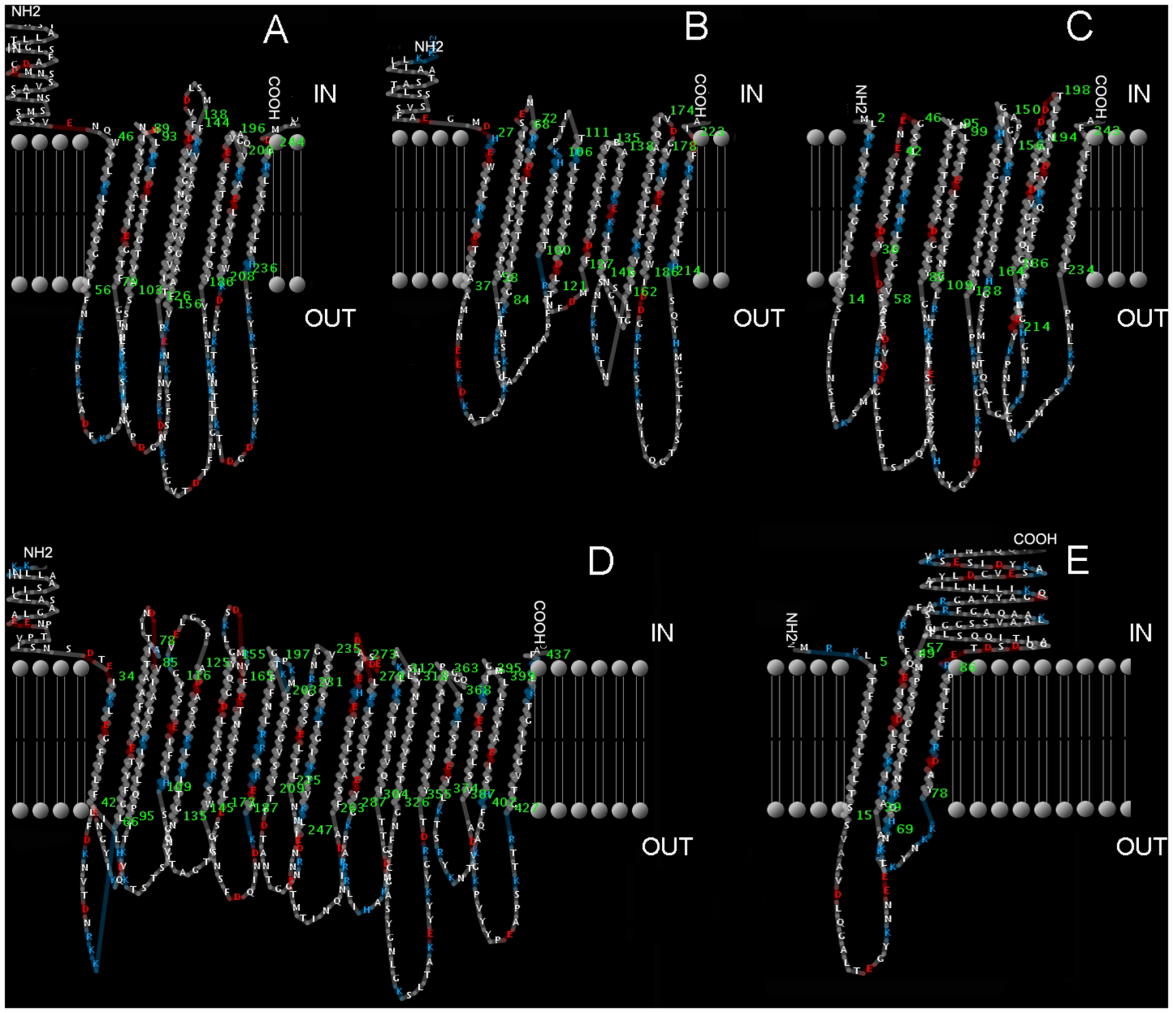
2-D transmembrane structures of surface-exposed proteins predicted using the PRED-TMBB server. The 2-D transmembrane structures of Adr1(A), Adr2 (B), OmpW (C), Porin_4 (D), and TolC (E) of *R. rickettsii* were designed by the transmembrane protein representation in 2 dimensions (TMRPres2D) tool according to their amino acid sequences, respectively. Coloring based on the charge and electric potential (assuming pH = 7): blue represents the negatively charged amino acids, red represents the positively charged amino acids, and gray indicates the neutral amino acids. IN means intracellular membrane, OUT means outer membrane, gray ball and double lines represent the lipid bilayer, the green numbers represent the amino acid ID inside or outside of the cell membrane; NH_2_ and COOH represent the amino and carboxy terminus, respectively.

The sequences were analyzed using the PRED-TMBB web server, and the transmembrane strands and the topology of β-barrel outer membrane proteins were predicted with the posterior decoding method. The discrimination scores of all the SEPs except GroES and DNA-binding protein were less than 2.965, which indicated that they were outer membrane proteins. Adr1 (Figure 2A) has 8 trans-membrane beta strands connected by 4 long annulations in the outer membrane and 3 short-chains in the inner membrane of rickettsiae. Adr2 (Figure 2B) and OmpW (Figure 2C) both have 10 trans-membrane beta strands connected by 5 long annulations in the outer membrane and 4 short-chains in the inner membrane. Porin_4 (Figure 2D) has 20 trans-membrane beta strands connected by 10 long annulations in the outer membrane and 9 short-chains in the inner membrane. TolC (Figure 2E) has 4 trans-membrane beta strands connected by 2 long annulations in the outer membrane and 1 short-chain in the inner membrane.

### Immunoblotting analysis of recombinant surface-exposed proteins

**Figure 3:**
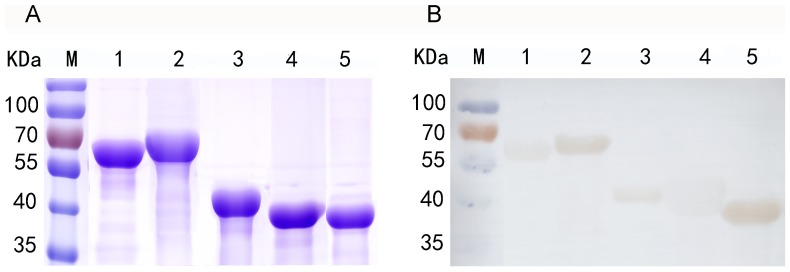
Immunoblotting analysis of surface-exposed proteins. (A) Five recombinant SEPs purified from *E. coli* cell lysate were separated by 12% SDS-PAGE and stained by G-250 Coomassie Brilliant Blue. (B) Immunoblotting analysis of SEPs: lane M, protein molecular mass markers; lanes 1 to 5, rTolC, rPorin_4, rAdr1, rOmpW, and rAdr2.

The purified recombinant proteins Adr1 (rAdr1, ∼43 kDa), rAdr2 (∼41 kDa), rOmpW (∼42 kDa), rPorin_4 (∼64 kDa), and rTolC (∼71 kDa) were separated by SDS-PAGE (Figure 3A). In the immunoblotting assay, each of the 5 proteins reacted with sera from *R. rickettsii*-infected mice, and the serological reaction of rAdr2 or rPorin_4 was stronger than that of rAdr1, rTolC, or rOmpW (Figure 3B).

### Immune protection elicited by surface-exposed proteins

**Figure 4:**
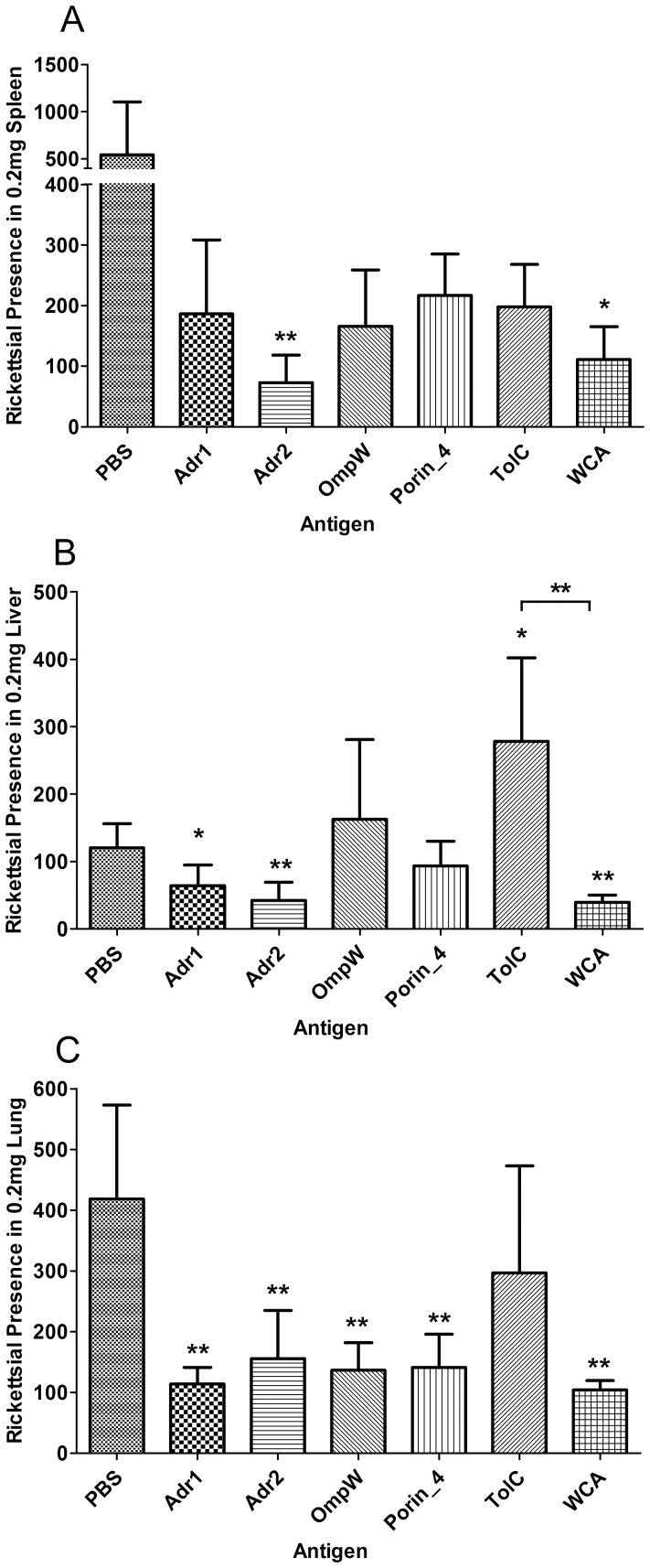
Protection against *R. rickettsii* infection induced by surface-exposed proteins. C3H/HeN mice (*n* = 5) were immunized three times with each rSEPs, WCA, or PBS, followed by a sublethal challenge with *R. rickettsii*. Five days after challenge, the mice were sacrificed and the rickettsial load in their spleens (A), livers (B), or lungs (C) was determined using *R. rickettsii*-specific qPCR. The rickettsial load is expressed as mean copies ± standard deviations, and the results were analyzed using *T* test or Wilcoxon two-sample test according to their normality and homogeneity of variance, *P<0.05 and **P<0.01. Horizontal lines indicate statistically significant differences between the WCA-immunized group and the rSEPs-immunized groups.

The C3H/HeN mice were immunized with each of the 5 rSEPs, and the immunized mice were challenged with viable *R. rickettsii* cells. Five days after challenge, the mice were sacrificed and rickettsial burdens in their spleens, livers, or lungs were determined by qPCR. The rickettsial load in spleen, liver, or lung of mice immunized with rAdr2 or WCA of *R. rickettsii* was significantly lower than that in mice mock-immunized with PBS. In contrast, the rickettsial load in spleen or liver and lung of mice immunized with rAdr1, rOmpW, or rPorin_4, but not with rTolC, was lower and significantly lower, respectively, compared with mock-immunized mice (Figure 4).

### Serum neutralization

**Figure 5:**
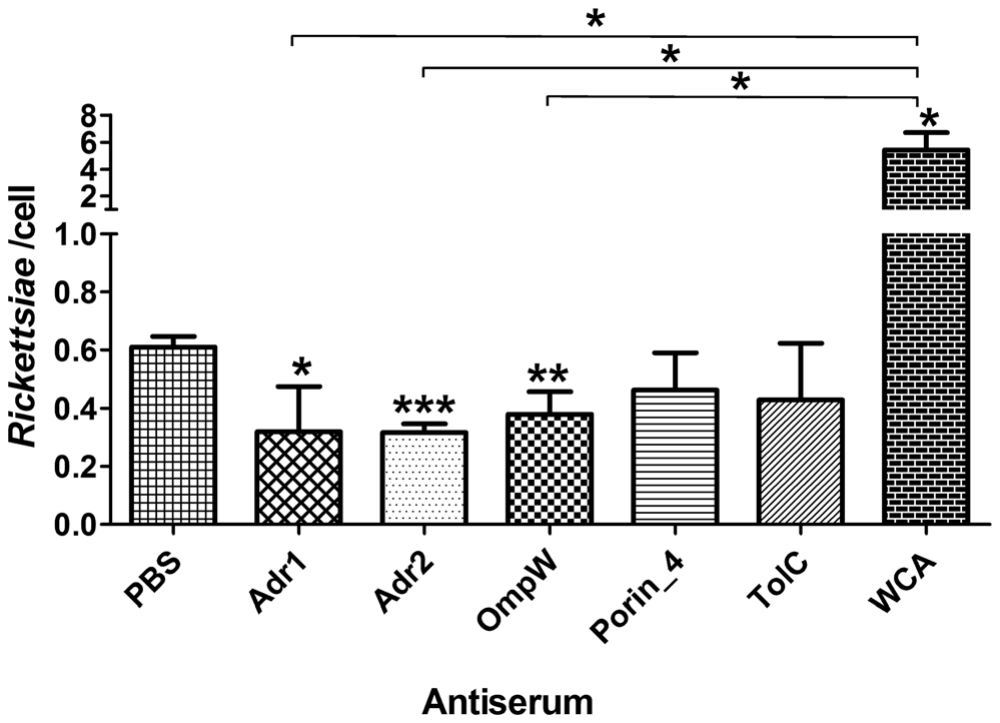
Neutralization of *R. rickettsii* by sera from mice immunized with surface-exposed proteins. Viable *R. rickettsii* organisms were incubated with naïve serum, anti-rAdr1, -rAdr2, -rOmpW, -rPorin_4, -rTolC, or -WCA (positive control) immune serum for 60 min. After incubation, the mixture was added into EA.hy 926 cells for 6 h. The total number of *R. rickettsii* that adhered to and invaded the host cells was determined using *R. rickettsii*-specific qPCR. The results were analyzed using the *T* test or Wilcoxon two-sample test according to their normality and homogeneity of variance. The statistically significant differences between anti-WCA immune serum and anti-rSEPs immune serum groups were indicated by horizontal lines. The values are presented as mean ± standard deviations (*n* = 3); *P<0.05, **P<0.01, and ***P<0.001.

Viable *R. rickettsii* organisms were incubated with various immunosera, and then the serum-treated organisms were used to infect vascular endothelial cells (host cells). After incubation host cells with the serum-treated organisms, the total amount of rickettsiae treated with rAdr1-, rAdr2-, or rOmpW-immunized sera was significantly less than that treated with sera from mice that had been mock-immunized with PBS (negative sera). In contrast, the total amount of rickettsiae treated with rPorin_4- or rTolC-immunized sera was less than that treated with negative sera, although this difference was not significant (Figure 5). Interestingly, the total amount of rickettsiae treated with WCA-immunized sera was significantly higher than that treated with rAdr1-, rAdr2-, rOmpW- or PBS-immunized serum.

### Detection of surface-exposed proteins by immuno-electron microscopic assay

**Figure 6:**
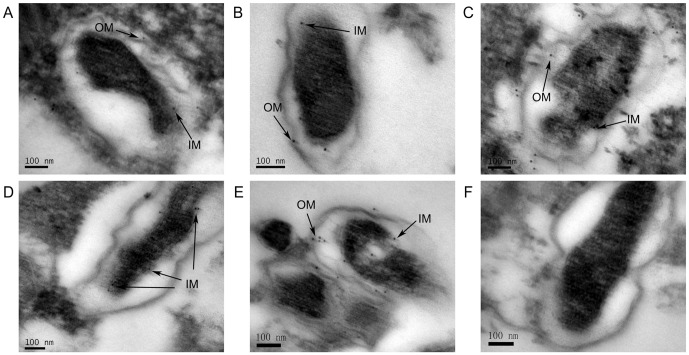
Detection of surface-exposed proteins using immuno-electron microscopic assay. Vero cells infected with *R. rickettsii* were incubated with anti-rAdr1 (A), -rAdr2 (B), -rOmpW (C), -rPorin_4 (D), -rTolC (E), or -PBS (F) serum and subsequently immunolabeled with colloidal gold particles (10 nm) using standard procedures. The cells were then observed by transmission electron microscope. The black arrows indicate target SEPs in the inner membrane (IM) and outer membrane (OM) of *R. rickettsii*. Bar  = 100 nm.

To further validate their location on the surface of rickettsiae, *R. rickettsii* cells were immunolabeled with colloidal gold using immuno-electron microscopic assay. As shown in Figure 6, *R. rickettsii* cells treated with rAdr1-, rAdr2-, rOmpW-, rPorin_4-, or rTolC-immunized sera, but not with negative sera, were decorated with gold particles. The particles were observed in both the outer and the inner membrane of *R. rickettsii* treated with rAdr1-, rAdr2-, rOmpW-, or rTolC-immunized sera, while only *R. rickettsii* treated with Porin_4- immunized sera displayed particles in the inner membrane.

## Discussion

*Rickettsia* spp. are obligate intracellular bacteria, and their surface molecules provide a crucial interface for their interactions with host cells [27]. SEPs may mediate rickettsial adherence to host cells and subsequent interaction with host cytosolic proteins, promoting rickettsial invasion, survival and replication in host cells [10]–[14]. In addition, rickettsial SEPs may be important antigens that efficiently activate immunocytes, including dendritic cells, NK cells, and/or T/B lymphocytes, to induce protection against rickettsial infection [15], [16], [28]–[30]. Characterization of *R. rickettsii* SEPs will provide an important foundation for further understanding the interactions between rickettsiae and hosts. In the present study, 10 major SEPs of *R. rickettsii* were identified using biotin-streptavidin affinity chromatography coupled with 2D-PAGE and ESI-MS/MS. The prediction of transmembrane strands and the topology of β-barrel outer membrane proteins suggested that all of the 10 SEPs except GroEL, GroES and DNA-binding protein were outer membrane proteins of *R. rickettsii*.

Among the 10 SEPs, OmpA and OmpB are well known major outer membrane proteins of *R. rickettsii* and function in rickettsial invasion of host cells [10], [31]–[33]. Both GroEL and GroES belong to the heat shock proteins family, which plays important roles in the folding, preventing aggregation and repairing misfolded or damaged proteins in *R. rickettsii* [34]. In addition to the cytoplasm, GroEL and/or GroES were also found on cell surfaces or in membrane fractions of *R. conorii* [35], *R. felis* [36], *R. parkeri* [37], and/or *R. typhi* [27]. DNA-binding protein has a strong adsorption capacity and tendency to small nucleotide fragments, which participates in NF-κB activation in response to *R. rickettsii*-induced expression of certain genes in vascular endothelial cells [38].

In the present study, we focused only on the newly identified SEPs (Adr1, Adr2, OmpW, Porin_4, and TolC) of *R. rickettsii*. Rickettsial adhesins Adr1 and Adr2 were respectively identified in two strong spots in the SEP profile of *R. rickettsii*. Both of these proteins have been previously recognized in *R. prowazekii* [39], *R. conorii* [40], and *R. heilongjiangensis* [16], and shown to participate in rickettsial adhesion and entry into the host cells [39]–[41] since antibody to Adr1 in *R. conorii* and antibody to Adr2 in *R. prowazekii* could efficiently inhibit rickettsia invasion of host cells [39], [41]. OmpW was previously identified in *R. heilongjiangensis* [16] and is typically localized to the outer membrane of Gram-negative bacteria and associated with bacterial resistance to various forms of environmental stress [42]. Porin_4 was identified in three spots (spots 17, 18 and 19) in the profile. This abundant protein is typically present as a trimer in the outer membrane of Gram-negative bacteria [43] and forms a relatively large water-filled channel and molecular filter, that allows the diffusion of hydrophilic molecules into the periplasmic space [44]. Additionally, porins are major outer membrane proteins that also serve as binding sites of phages and bacteriocins [44]. TolC, which was identified in one spot close to porin_4 in the SEP profile, is usually present as a trimeric channel across the inner and outer membrane of Gram-negative bacterial and is required for the export of virulence proteins and toxic compounds [45], [46]. In *Rickettsia typhi*, TolC was found to be the component of the putative type I secretion system, functioning in secretion of rickettsia ankyrin repeat protein 1 (RARP-1) across the outer membrane of *R. typhi* into the cytoplasm of mammalian cells [47]. Comparison of Adr1 (Figure S1), Adr2 (Fig S2), OmpW (Figure S3), Porin_4 (Figure S4), and TolC (Figure S5) amino acid sequences from SFG rickettsiae showed that all of them were high conservative among SFG rickettsiae (with an amino acid similarity of 87%∼99%) except *R. akari*, *R. australis*, and *R. helvetica* (Table S2).

The 5 novel SEPs of *R. rickettsii* were found to be membrane-spanning proteins. To further define their location, *R. rickettsii* organisms were stained with specific immunosera to each SEP using immunoelectron microscopy, which has been successfully applied to determine the surface expression of RickA in *R. raoultii* [48]. All of the SEPs except Porin_4 were present in both the inner and outer membrane, while Porin_4 was observed only in the inner membrane of *R. rickettsii* based on immuno-electron microscopic assay despite its previous identification in the outer membrane of Gram-negative bacteria [43]. Immuno-electron microscopic assay revealed that each SEPs had a punctate distribution on the surface of *R. rickettsii* rather than a diffuse distribution, which suggested that they were located in different regions of the rickettsial membrane. This localization might be associated with their special function (s), such as attachment, motility, molecular transport, and/or conjugation.

SFG rickettsiae express a family of Sca (surface cell antigen) proteins [9]. Members of this family have modular structures, including an N-terminal signal sequence, a central passenger peptide, and a C-terminal “translocation module” (β-peptide) [49]. Following translation, the peptide is initially secreted across the inner membrane according to information present in the N-terminal signal sequence. The C-terminal peptide is then inserted into the outer membrane to form a barrel-rich transmembrane pore through which the passenger peptide passes, exposing the passenger peptide to the extracellular environment [11]. Based on these features, the 5 novel SEPs of *R. rickettsii* should belong to the Sca family.

Although previous studies have demonstrated that antibodies against Adr1 in *R. conorii* and Adr2 in *R. prowazekii* can prevent rickettsiae from entering host cells [39], [41], the functions of the 5 novel SEPs of *R. rickettsii* remain to be described. To assay the potential effects of specific antibodies against these SEPs on the interaction of *R. rickettsii* with vascular endothelial cells, sera from mice immunized with each of the 5 rSEPs were applied to neutralize *R. rickettsii in vitro*. Our results showed that the specific antibodies in sera from mice immunized with any of the 5 rSEPs could reduce rickettsial invasion of vascular endothelial cells, and this reduction by sera from rAdr1-, rAdr2-, or rOmpW-immunized mice was significant. We postulate that the 5 novel SEPs, particularly Adr1, Adr2, and OmpW, are involved in the interaction of *R. rickettsii* with vascular endothelial cells due to the potential of the specific antibodies to prevent the rickettsial surface molecules from interaction with the corresponding surface molecules on host cells. Additionally, sera from mice immunized with *R. rickettsii* WCA markedly enhanced rickettsial invasion of vascular endothelial cells, which has been previously observed in neutralization assays with sera from mice immunized with *R. conorii* or *R. heilongjiangensis* [50], [51]. It has been postulated that antibodies against numerous rickettsial surface molecules in WCA-immunized sera can interact with multiple Fc receptors expressed by vascular endothelial cells, potentially promoting rickettsial entrance into host cells [50].

In addition, SEPs may be important antigen molecules to mediate interaction between rickettsiae and hosts cells [52], playing a key role in immune responses against rickettsial infection [16]. To validate their capacity to elicit specific immune protection, these rSEPs were used to immunize mice, which were subsequently challenged with viable *R. rickettsii* cells. Our results showed that the rickettsial load in lung, spleen, or liver of mice immunized with rAdr2 and that in lung of mice immunized with rAdr1, rOmpW, or rPorin_4, but not rTolC, were significantly lower than of the rickettsial load in mock-immunized mice. The lung is the most important target organ of *R. rickettsii*, and thus, the significant protection of the lung conferred by Adr1, Adr2, OmpW, and Porin_4, suggests they are protective antigens. Surprisingly, the protective efficacy of rAdr2 was similar to that of WCA of *R. rickettsii*. This result firmly indicates that Adr2 is a candidate vaccine against *R. rickettsii* infection.

Discontinued RMSF vaccines derived from killed rickettsiae purified from *R. rickettsii*-infected ticks or embryonated eggs [53] were relatively ineffective and adversely reactive in preventing *R. rickettsii* infection in humans [54]. Therefore, there have been efforts to develop a safe and effective subunit vaccine against RMSF. The protective antigens of *R. rickettsii*, identified in the present and in the previous studies, or their antigenic epitopes, particularly the immunodominant epitopes of T and B cells, may be combined to develop more efficient subunit vaccines of RMSF.

In conclusion, the 5 novel SEPs (Adr1, Adr2, OmpW, Porin_4, and TolC) were localized to the outer and/or inner membrane of *R. rickettsii* by immuno-electron microscopic assay. All of these rSEPs except rTolC conferred significant protection against *R. rickettsii* in the lungs of mice, and rAdr2 induced more efficient protection similarly to *R. rickettsii* WCA. These findings suggested that Adr1, Adr2, OmpW, and Porin_4 were protective antigens. Sera from mice immunized with each of the rSEPs reduced invasion of *R. rickettsii* into vascular endothelial cells, and this reduction by sera from rAdr1-, rAdr2-, or rOmpW-immunized mice was significant. These results indicated that the novel SEPs, particularly Adr1, Adr2, and OmpW, were involved in the interaction of *R. rickettsii* with vascular endothelial cells.

## Supporting Information

Figure S1 **Comparison analysis of Adr1 amino acid sequences from spotted fever group rickettsiae.** Adr1 amino sequences between *R. rickettsii* (list on the top line) and other spotted fever group rickettsiae (list under the *R. rickettsii* line) were compared by CLC Genomic Workbench V3.6.1 software (CLC BIO Inc., Aarhus, Denmark). NCBI accession numbers of Adr1 in SFG rickettsiae as follows: *Rickettsia rickettsii* str. Sheila Smith, ABV76850.1; *Rickettsia africae* str. ESF-5, YP_002845692.1; *Rickettsia akari* str. Hartford, YP_001494015.1; *Rickettsia amblyommii* str. GAT-30V, YP_005365990.1; *Rickettsia australis* str. Cutlack, YP_005414527.1; *Rickettsia conorii* str. Malish 7, NP_360918.1; *Rickettsia heilongjiangensis* str. 054, YP_004764920.1; *Rickettsia Helvetica*, WP_010421009.1; *Rickettsia honei*, WP_016917657.1, *Rickettsia japonica* str. YH, YP_004885269.1; *Rickettsia massiliae* str. MTU5, YP_001499799.1; *Rickettsia montanensis* str. OSU 85-930, YP_005391649.1; *Rickettsia parkeri* str. Portsmouth, YP_005393487.1; *Rickettsia peacockii* str. Rustic, YP_002916697.1; *Rickettsia rhipicephali* str. 3-7-female6-CWPP, YP_005391036.1; *Rickettsia sibirica* 246, WP_004997220.1; *Rickettsia slovaca* str. D-CWPP, YP_005066302.1.
(TIF)

Figure S2 **Comparison analysis of Adr2 amino acid sequences from spotted fever group rickettsiae.** Adr2 amino sequences between *R. rickettsii* (list on the top line) and other spotted fever group rickettsiae (list under the *R. rickettsii* line) were compared by CLC Genomic Workbench V3.6.1 software (CLC BIO Inc., Aarhus, Denmark). NCBI accession numbers of Adr2 in SFG rickettsiae as follows: *Rickettsia rickettsii* str. Sheila Smith, ABV76851.1; *Rickettsia africae* str. ESF-5, YP_002845693.1; *Rickettsia akari* str. Hartford, YP_001494016.1; *Rickettsia amblyommii* str. GAT-30V, YP_005365991.1; *Rickettsia australis* str. Cutlack, YP_005414526.1; *Rickettsia conorii* str. Malish 7, NP_360919.1; *Rickettsia heilongjiangensis* str. 054, YP_004764921.1; *Rickettsia Helvetica*, WP_010421007.1; *Rickettsia honei*, WP_016917656.1, *Rickettsia japonica* str. YH, YP_004885270.1; *Rickettsia massiliae* str. MTU5, YP_005302541.1; *Rickettsia montanensis* str. OSU 85-930, YP_005391650.1; *Rickettsia parkeri* str. Portsmouth, YP_005393488.1; *Rickettsia peacockii* str. Rustic, YP_002916696.1; *Rickettsia rhipicephali* str. 3-7-female6-CWPP, YP_005391037.1; *Rickettsia sibirica* 246, WP_004997218.1; *Rickettsia slovaca* str. D-CWPP, YP_005066303.1.
(TIF)

Figure S3 **Comparison analysis of OmpW amino acid sequences from spotted fever group rickettsiae.** OmpW amino sequences between *R. rickettsii* (list on the top line) and other spotted fever group rickettsiae (list under the *R. rickettsii* line) were compared by CLC Genomic Workbench V3.6.1 software (CLC BIO Inc., Aarhus, Denmark). NCBI accession numbers of OmpW in SFG rickettsiae as follows: *Rickettsia rickettsii* str. Sheila Smith, ABV75714.1; *Rickettsia africae* str. ESF-5, YP_002844819.1; *Rickettsia akari* str. Hartford, YP_001492955.1; *Rickettsia amblyommii* str. GAT-30V, YP_005364912.1; *Rickettsia australis* str. Cutlack, YP_005414414.1; *Rickettsia conorii* str. Malish 7, NP_359742.1; *Rickettsia heilongjiangensis* str. 054, YP_004763826.1; *Rickettsia Helvetica*, WP_010420430.1; *Rickettsia honei*, WP_016917580.1, *Rickettsia japonica* str. YH, YP_004884443.1; *Rickettsia massiliae* str. MTU5, YP_001498965.1; *Rickettsia montanensis* str. OSU 85-930, YP_005391832.1; *Rickettsia parkeri* str. Portsmouth, YP_005392367.1; *Rickettsia peacockii* str. Rustic, YP_002916393.1; *Rickettsia rhipicephali* str. 3-7-female6-CWPP, YP_005389961.1; *Rickettsia sibirica* 246, WP_004996776.1; *Rickettsia slovaca* str. D-CWPP, YP_005065351.1.
(TIF)

Figure S4 **Comparison analysis of Porin_4 amino acid sequences from spotted fever group rickettsiae.** Porin_4 amino sequences between *R. rickettsii* (list on the top line) and other spotted fever group rickettsiae (list under the *R. rickettsii* line) were compared by CLC Genomic Workbench V3.6.1 software (CLC BIO Inc., Aarhus, Denmark). NCBI accession numbers of Porin_4 in SFG rickettsiae as follows: *Rickettsia rickettsii* str. Sheila Smith, ABV75707.1; *Rickettsia africae* str. ESF-5, YP_002844812.1; *Rickettsia akari* str. Hartford, YP_001492948.1; *Rickettsia amblyommii* str. GAT-30V, YP_005364919.1; *Rickettsia australis* str. Cutlack, YP_005414407.1; *Rickettsia conorii* str. Malish 7, NP_359735.1; *Rickettsia heilongjiangensis* str. 054, YP_004763819.1; *Rickettsia Helvetica*, WP_010420474.1; *Rickettsia honei*, WP_016917574.1, *Rickettsia japonica* str. YH, YP_004884436.1; *Rickettsia massiliae* str. MTU5, YP_001498958.1; *Rickettsia montanensis* str. OSU 85-930, YP_005391825.1; *Rickettsia parkeri* str. Portsmouth, YP_005392360.1; *Rickettsia peacockii* str. Rustic, YP_002916386.1; *Rickettsia rhipicephali* str. 3-7-female6-CWPP, YP_005389954.1; *Rickettsia sibirica* 246, WP_004996798.1; *Rickettsia slovaca* str. D-CWPP, YP_005065344.1.
(TIF)

Figure S5 **Comparison analysis of TolC amino acid sequences from spotted fever group rickettsiae.** TolC amino sequences between *R. rickettsii* (list on the top line) and other spotted fever group rickettsiae (list under the *R. rickettsii* line) were compared by CLC Genomic Workbench V3.6.1 software (CLC BIO Inc., Aarhus, Denmark). NCBI accession numbers of TolC in SFG rickettsiae as follows: *Rickettsia rickettsii* str. Sheila Smith, ABV75915.1; *Rickettsia africae* str. ESF-5, YP_002844973.1; *Rickettsia akari* str. Hartford, YP_001493153.1; *Rickettsia amblyommii* str. GAT-30V, YP_005365053.1; *Rickettsia australis* str. Cutlack, YP_005415374.1; *Rickettsia conorii* str. Malish 7, NP_359943.1; *Rickettsia heilongjiangensis* str. 054, YP_004764024.1; *Rickettsia Helvetica*, WP_010423612.1; *Rickettsia honei*, WP_016917019.1, *Rickettsia japonica* str. YH, YP_004884596.1; *Rickettsia massiliae* str. MTU5, YP_001499115.1; *Rickettsia montanensis* str. OSU 85-930, YP_005392010.1; *Rickettsia parkeri* str. Portsmouth, YP_005392559.1; *Rickettsia peacockii* str. Rustic, YP_002916267.1; *Rickettsia rhipicephali* str. 3-7-female6-CWPP, YP_005390149.1; *Rickettsia sibirica* 246, WP_004996393.1; *Rickettsia slovaca* str. D-CWPP, YP_005065520.1.
(TIF)

Table S1 **Primer sequences and cleavage sites of surface-exposed proteins.**
(DOCX)

Table S2 **Matching ratio of five novel surface-exposed proteins in spotted fever group rickettsiae.**
(DOCX)
