# Supplementary figures and images for: Identification of Novel Surface-Exposed Proteins of Rickettsia rickettsii by Affinity Purification and Proteomics
Source: PLoS One. 2014 Jun 20;9(6):e100253. doi: 10.1371/journal.pone.0100253 (PMC4065002; doi:10.1371/journal.pone.0100253)

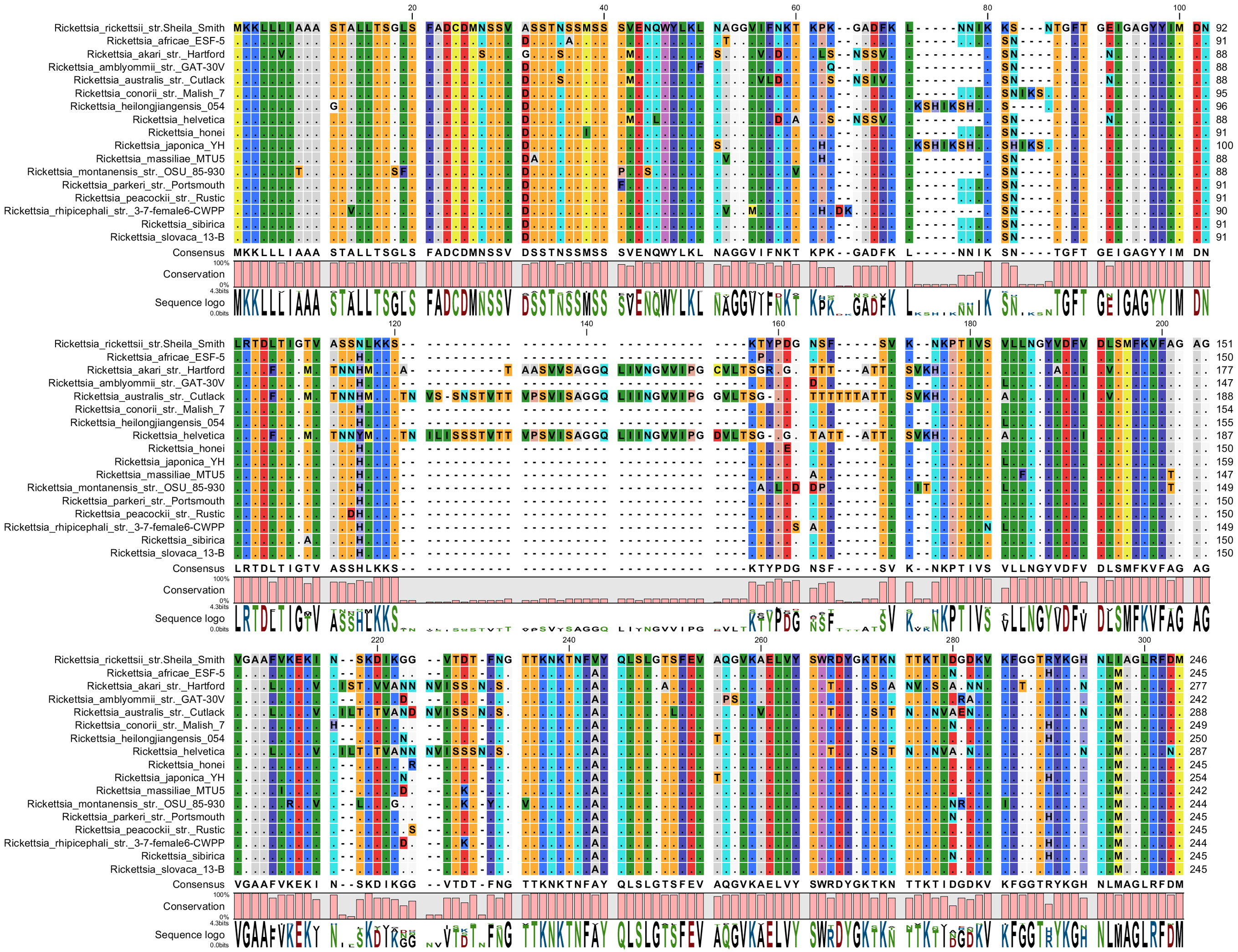

Supplement: Figure S1 — Comparison analysis of Adr1 amino acid sequences from spotted fever group rickettsiae. Adr1 amino sequences between R. rickettsii (list on the top line) and other spotted fever group rickettsiae (list under the R. rickettsii line) were compared by CLC Genomic Workbench V3.6.1 software (CLC BIO Inc., Aarhus, Denmark). NCBI accession numbers of Adr1 in SFG rickettsiae as follows: Rickettsia rickettsii str. Sheila Smith, ABV76850.1; Rickettsia africae str. ESF-5, YP_002845692.1; Rickettsia akari str. Hartford, YP_001494015.1; Rickettsia amblyommii str. GAT-30V, YP_005365990.1; Rickettsia australis str. Cutlack, YP_005414527.1; Rickettsia conorii str. Malish 7, NP_360918.1; Rickettsia heilongjiangensis str. 054, YP_004764920.1; Rickettsia Helvetica, WP_010421009.1; Rickettsia honei, WP_016917657.1, Rickettsia japonica str. YH, YP_004885269.1; Rickettsia massiliae str. MTU5, YP_001499799.1; Rickettsia montanensis str. OSU 85-930, YP_005391649.1; Rickettsia parkeri str. Portsmouth, YP_005393487.1; Rickettsia peacockii str. Rustic, YP_002916697.1; Rickettsia rhipicephali str. 3-7-female6-CWPP, YP_005391036.1; Rickettsia sibirica 246, WP_004997220.1; Rickettsia slovaca str. D-CWPP, YP_005066302.1. (TIF) [file pone.0100253.s001.tif]

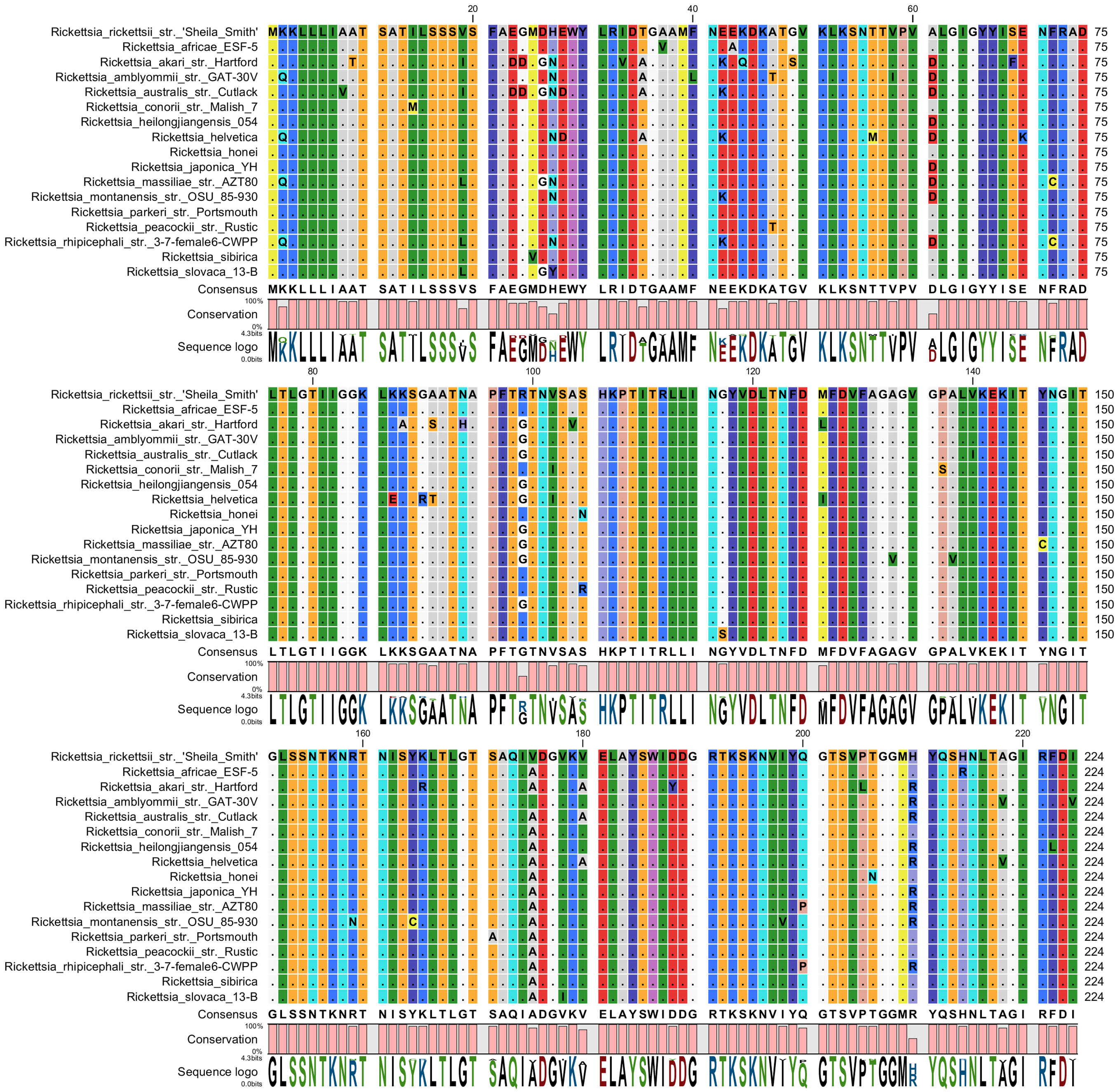

Supplement: Figure S2 — Comparison analysis of Adr2 amino acid sequences from spotted fever group rickettsiae. Adr2 amino sequences between R. rickettsii (list on the top line) and other spotted fever group rickettsiae (list under the R. rickettsii line) were compared by CLC Genomic Workbench V3.6.1 software (CLC BIO Inc., Aarhus, Denmark). NCBI accession numbers of Adr2 in SFG rickettsiae as follows: Rickettsia rickettsii str. Sheila Smith, ABV76851.1; Rickettsia africae str. ESF-5, YP_002845693.1; Rickettsia akari str. Hartford, YP_001494016.1; Rickettsia amblyommii str. GAT-30V, YP_005365991.1; Rickettsia australis str. Cutlack, YP_005414526.1; Rickettsia conorii str. Malish 7, NP_360919.1; Rickettsia heilongjiangensis str. 054, YP_004764921.1; Rickettsia Helvetica, WP_010421007.1; Rickettsia honei, WP_016917656.1, Rickettsia japonica str. YH, YP_004885270.1; Rickettsia massiliae str. MTU5, YP_005302541.1; Rickettsia montanensis str. OSU 85-930, YP_005391650.1; Rickettsia parkeri str. Portsmouth, YP_005393488.1; Rickettsia peacockii str. Rustic, YP_002916696.1; Rickettsia rhipicephali str. 3-7-female6-CWPP, YP_005391037.1; Rickettsia sibirica 246, WP_004997218.1; Rickettsia slovaca str. D-CWPP, YP_005066303.1. (TIF) [file pone.0100253.s002.tif]

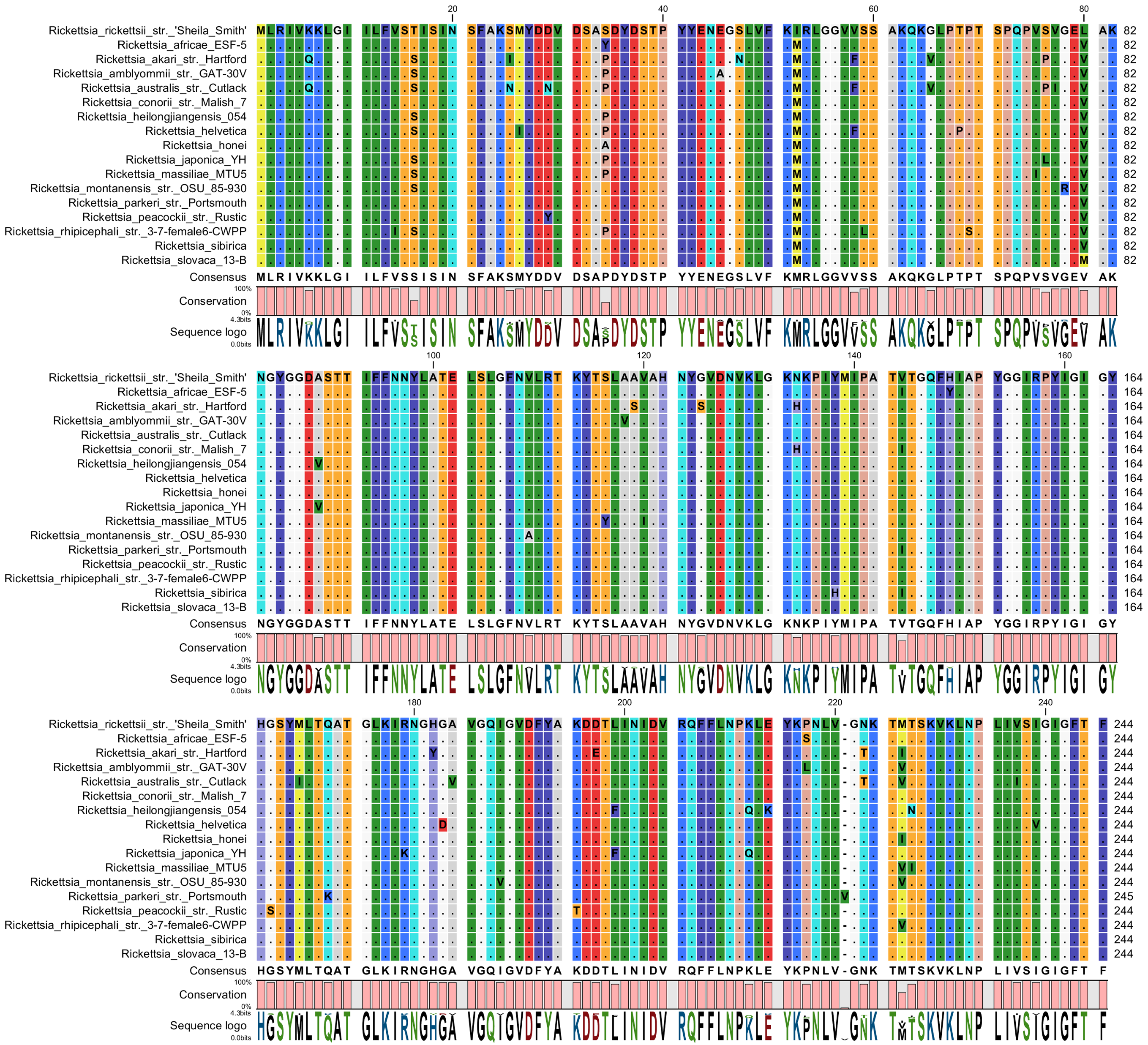

Supplement: Figure S3 — Comparison analysis of OmpW amino acid sequences from spotted fever group rickettsiae. OmpW amino sequences between R. rickettsii (list on the top line) and other spotted fever group rickettsiae (list under the R. rickettsii line) were compared by CLC Genomic Workbench V3.6.1 software (CLC BIO Inc., Aarhus, Denmark). NCBI accession numbers of OmpW in SFG rickettsiae as follows: Rickettsia rickettsii str. Sheila Smith, ABV75714.1; Rickettsia africae str. ESF-5, YP_002844819.1; Rickettsia akari str. Hartford, YP_001492955.1; Rickettsia amblyommii str. GAT-30V, YP_005364912.1; Rickettsia australis str. Cutlack, YP_005414414.1; Rickettsia conorii str. Malish 7, NP_359742.1; Rickettsia heilongjiangensis str. 054, YP_004763826.1; Rickettsia Helvetica, WP_010420430.1; Rickettsia honei, WP_016917580.1, Rickettsia japonica str. YH, YP_004884443.1; Rickettsia massiliae str. MTU5, YP_001498965.1; Rickettsia montanensis str. OSU 85-930, YP_005391832.1; Rickettsia parkeri str. Portsmouth, YP_005392367.1; Rickettsia peacockii str. Rustic, YP_002916393.1; Rickettsia rhipicephali str. 3-7-female6-CWPP, YP_005389961.1; Rickettsia sibirica 246, WP_004996776.1; Rickettsia slovaca str. D-CWPP, YP_005065351.1. (TIF) [file pone.0100253.s003.tif]

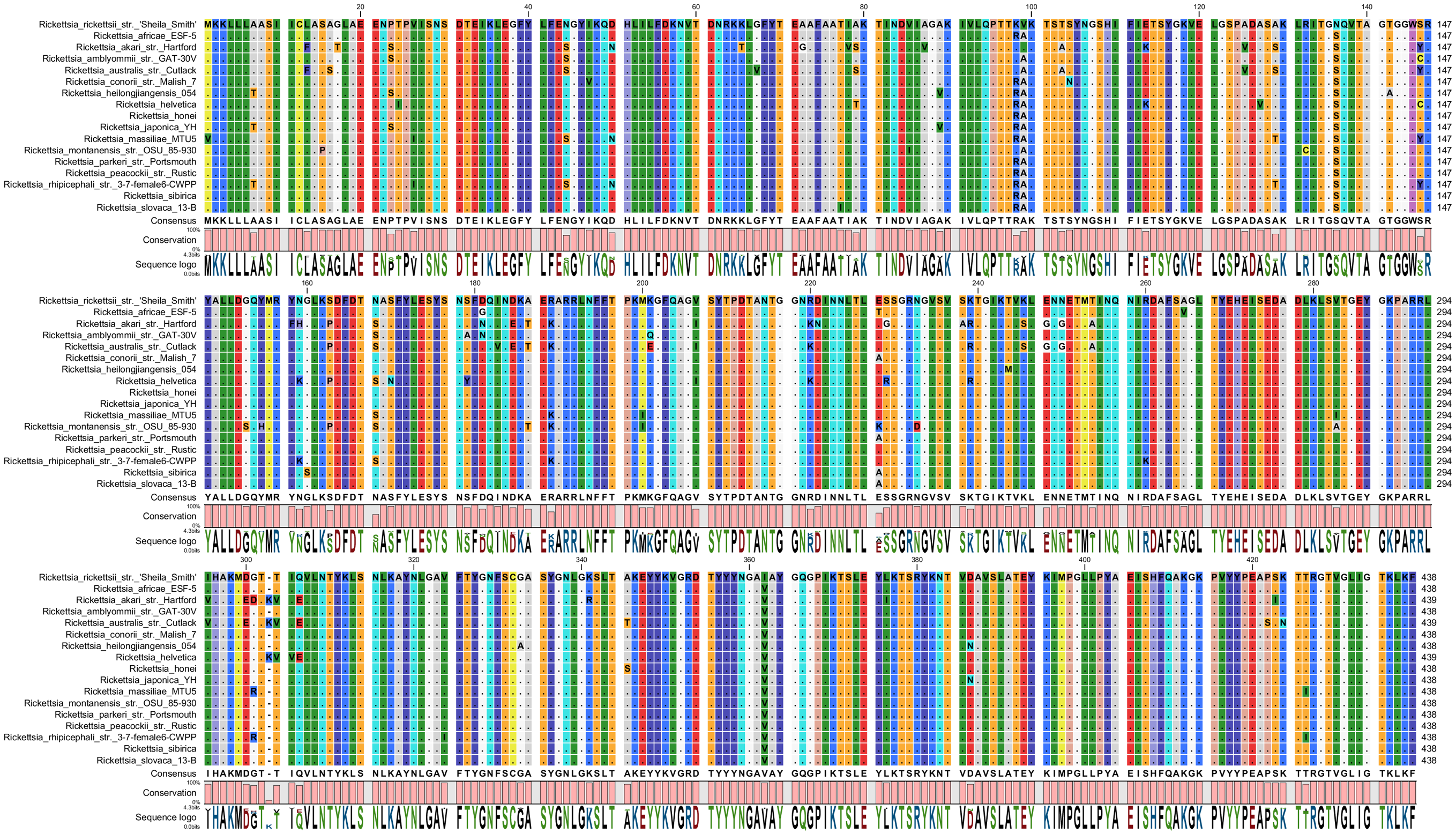

Supplement: Figure S4 — Comparison analysis of Porin_4 amino acid sequences from spotted fever group rickettsiae. Porin_4 amino sequences between R. rickettsii (list on the top line) and other spotted fever group rickettsiae (list under the R. rickettsii line) were compared by CLC Genomic Workbench V3.6.1 software (CLC BIO Inc., Aarhus, Denmark). NCBI accession numbers of Porin_4 in SFG rickettsiae as follows: Rickettsia rickettsii str. Sheila Smith, ABV75707.1; Rickettsia africae str. ESF-5, YP_002844812.1; Rickettsia akari str. Hartford, YP_001492948.1; Rickettsia amblyommii str. GAT-30V, YP_005364919.1; Rickettsia australis str. Cutlack, YP_005414407.1; Rickettsia conorii str. Malish 7, NP_359735.1; Rickettsia heilongjiangensis str. 054, YP_004763819.1; Rickettsia Helvetica, WP_010420474.1; Rickettsia honei, WP_016917574.1, Rickettsia japonica str. YH, YP_004884436.1; Rickettsia massiliae str. MTU5, YP_001498958.1; Rickettsia montanensis str. OSU 85-930, YP_005391825.1; Rickettsia parkeri str. Portsmouth, YP_005392360.1; Rickettsia peacockii str. Rustic, YP_002916386.1; Rickettsia rhipicephali str. 3-7-female6-CWPP, YP_005389954.1; Rickettsia sibirica 246, WP_004996798.1; Rickettsia slovaca str. D-CWPP, YP_005065344.1. (TIF) [file pone.0100253.s004.tif]

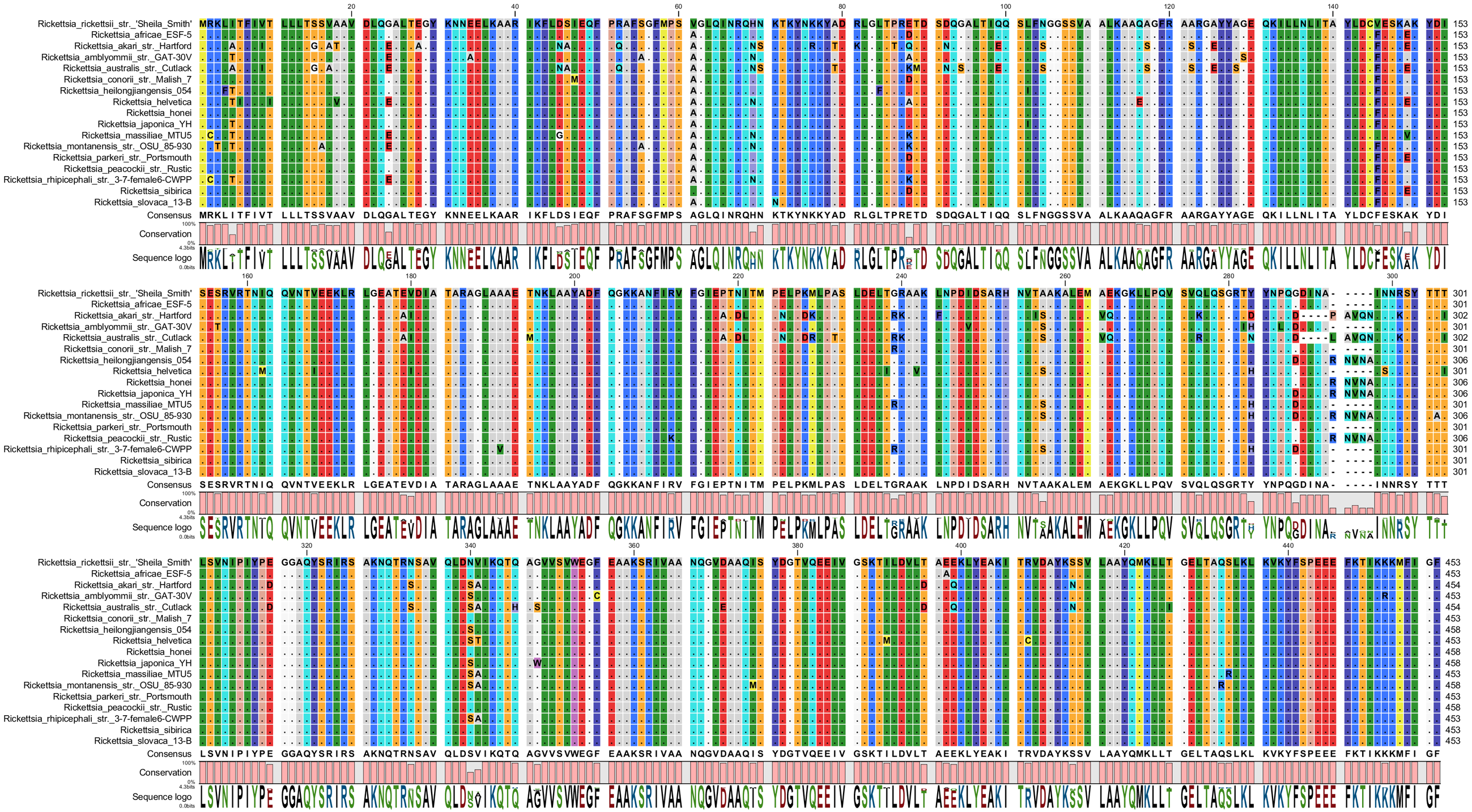

Supplement: Figure S5 — Comparison analysis of TolC amino acid sequences from spotted fever group rickettsiae. TolC amino sequences between R. rickettsii (list on the top line) and other spotted fever group rickettsiae (list under the R. rickettsii line) were compared by CLC Genomic Workbench V3.6.1 software (CLC BIO Inc., Aarhus, Denmark). NCBI accession numbers of TolC in SFG rickettsiae as follows: Rickettsia rickettsii str. Sheila Smith, ABV75915.1; Rickettsia africae str. ESF-5, YP_002844973.1; Rickettsia akari str. Hartford, YP_001493153.1; Rickettsia amblyommii str. GAT-30V, YP_005365053.1; Rickettsia australis str. Cutlack, YP_005415374.1; Rickettsia conorii str. Malish 7, NP_359943.1; Rickettsia heilongjiangensis str. 054, YP_004764024.1; Rickettsia Helvetica, WP_010423612.1; Rickettsia honei, WP_016917019.1, Rickettsia japonica str. YH, YP_004884596.1; Rickettsia massiliae str. MTU5, YP_001499115.1; Rickettsia montanensis str. OSU 85-930, YP_005392010.1; Rickettsia parkeri str. Portsmouth, YP_005392559.1; Rickettsia peacockii str. Rustic, YP_002916267.1; Rickettsia rhipicephali str. 3-7-female6-CWPP, YP_005390149.1; Rickettsia sibirica 246, WP_004996393.1; Rickettsia slovaca str. D-CWPP, YP_005065520.1. (TIF) [file pone.0100253.s005.tif]
