# Supplementary material for: Identification of Novel Surface-Exposed Proteins of Rickettsia rickettsii by Affinity Purification and Proteomics
Source: PLoS One. 2014 Jun 20;9(6):e100253. doi: 10.1371/journal.pone.0100253 (PMC4065002; doi:10.1371/journal.pone.0100253)
Supplement: Table S1 — Primer sequences and cleavage sites of surface-exposed proteins. (DOCX) [file pone.0100253.s006.docx]

| Primer name | Cleaving enzyme | cleavage sites | Sequence（5'→3'） | Gene length (bp) |
| --- | --- | --- | --- | --- |
| *adr1 F* | BamHI | 34 | GGAGGATCCTTAATAGCAGCTGCAAGCAC | 699 |
| *adr1 R* | XhoI | 732 | GGACTCGAGCATATCAAATCTTAATCCTG | 699 |
| *adr2 F* | BamHI | 13 | GCGGATCCCTTTTAATAGCTGCTAC | 654 |
| *adr2 R* | XhoI | 666 | GGCTCGAGAAATCTTATACCGGCTG | 654 |
| *ompW F* | BamHI | 25 | GGGGATCCGGGATAATTTTGTTTG | 660 |
| *ompW R* | XhoI | 684 | GGCTCGAGTTTAGAGGTCATGGTTTT | 660 |
| *porin_4 F* | BamHI | 25 | GGGGATCCAGTATTATTTGCCTTGC | 1266 |
| *porin_4 R* | XhoI | 1290 | GGCTCGAGACCAACAGTACCTCGT | 1266 |
| *tolC F* | BamHI | 79 | CCGGATCCACTGAAGGGTATAAGAA | 1248 |
| *tolC R* | SalI | 1326 | CCGTCGACAAACTCTTCTTCAGGACTA | 1248 |
