# Supplementary material for: Identification of Novel Surface-Exposed Proteins of Rickettsia rickettsii by Affinity Purification and Proteomics
Source: PLoS One. 2014 Jun 20;9(6):e100253. doi: 10.1371/journal.pone.0100253 (PMC4065002; doi:10.1371/journal.pone.0100253)
Supplement: Table S2 — Matching ratio of five novel surface-exposed proteins in spotted fever group rickettsiae. (DOCX) [file pone.0100253.s007.docx]

| Rickettsia species^*^ | Matching Ratio^#^ (%) | | | | |
| --- | --- | --- | --- | --- | --- |
|  | Adr1 | Adr2 | OmpW | Porin_4 | TolC |
| *Rickettsia rickettsii* str. Sheila Smith | 100 | 100 | 100 | 100 | 100 |
| *Rickettsia africae* str. ESF-5 | 96 | 98 | 98 | 98 | 99 |
| *Rickettsia akari* str. Hartford | 65 | 89 | 93 | 90 | 87 |
| *Rickettsia amblyommii* str. GAT-30V | 93 | 95 | 97 | 98 | 96 |
| *Rickettsia australis* str. Cutlack | 64 | 93 | 93 | 92 | 87 |
| *Rickettsia conorii* str. Malish 7 | 96 | 98 | 98 | 98 | 99 |
| *Rickettsia heilongjiangensis* str. 054 | 94 | 98 | 96 | 97 | 97 |
| *Rickettsia helvetica* | 65 | 92 | 96 | 95 | 95 |
| *Rickettsia honei* | 97 | 99 | 98 | 99 | 98 |
| *Rickettsia japonica* str. YH | 92 | 98 | 96 | 98 | 97 |
| *Rickettsia massiliae* str. MTU5 | 93 | 96 | 96 | 96 | 97 |
| *Rickettsia montanensis* str. OSU 85-930 | 87 | 95 | 97 | 96 | 95 |
| *Rickettsia parkeri* str. Portsmouth | 97 | 99 | 98 | 99 | 99 |
| *Rickettsia peacockii* str. Rustic | 97 | 99 | 98 | 99 | 98 |
| *Rickettsia rhipicephali* str. 3-7-female6-CWPP | 92 | 96 | 97 | 96 | 97 |
| *Rickettsia sibirica* 246 | 97 | 99 | 98 | 99 | 99 |
| *Rickettsia slovaca* str. D-CWPP | 98 | 97 | 99 | 99 | 99 |

* The spotted fever group corresponds to the *R. rickettsii* group (gr.), *R. massiliae* gr., *R. Helvetica* gr., and *R. Akari* gr. The classification of Rickettsia spp. is determined by Merhej Vicky & Raoult Didier (Biological Reviews, Volume 86, Number 2, May 2011 , pp. 379-405(27)).

# Indicates the matching ratio of *R. rickettsii* and each rickettsia among 16 spotted fever group rickettsiae.
